# Supplementary material for: Identification of photoperiod-induced specific miRNAs in the adrenal glands of Sunite sheep (Ovis aries)
Source: Front Vet Sci. 2022 Jul 22;9:888207. doi: 10.3389/fvets.2022.888207 (PMC9354845; doi:10.3389/fvets.2022.888207)
Supplement: Supplementary file 1 [file Data_Sheet_1.ZIP › Supplementary file/miRDeep2 file.zip!/miRDeep2 file/Know_Structure/LP42A1/oar-mir-543.pdf]

miRBase precursor : oar-mir-543
Total read count : 7094
oar-miR-543-5p read count : 5
oar-miR-543-3p read count : 7059
remaining reads : 30

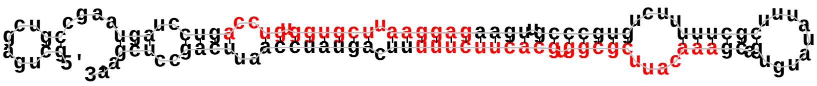

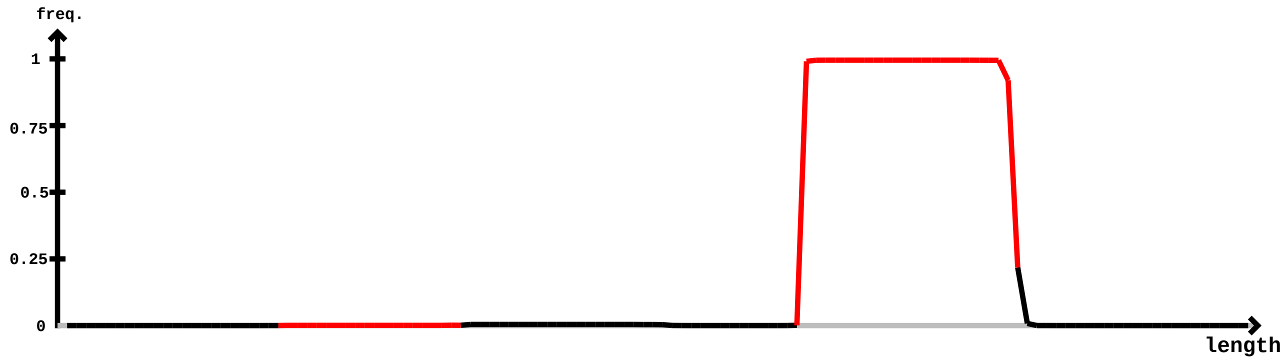


```
                                                                           oar-miR-543-3p
                   oar-miR-543-5p
5'- gcugagcugccgaaugauccugaccuguggugcuuaaggagaaguugcccgugucuuuuucgcuuuauaugugacgaaacauucgcggugcacuucuuuuucaguauccaauucagccucgaa  -3'   exp
    ((......)).....((..((((..((.((((((.((((((((((.(((((((....((((((.........).)))))....))))).)))))))))))).))))))))..))))..))...   reads   mm
    ....................ugaccuguggugcuuaagga...................................................................................   1       0
    ......................accuguggugcuuaagA....................................................................................   1       1
    ......................accuguggugcuuaagga...................................................................................   2       0
    ......................Gccuguggugcuuaagga...................................................................................   1       1
    .......................................agaaguugcccgugucuuuu................................................................   2       0
    .......................................agaaguugcccgugucuuuuuc..............................................................   2       0
    .......................................agaaguugcccgugucuuuuucg.............................................................   2       0
    ........................................gaaguugcccgugucuuuuucg.............................................................   2       0
    .........................................aaguugcccgugucuuuuuc..............................................................   1       0
    .........................................aaguugcccgugucuuuuucg.............................................................   18      0
    .........................................aaguugcccgugucuuuuucgc............................................................   2       0
    .........................................aaguugcccgugucuuuuucgcu...........................................................   1       0
    ..........................................................................cgaaacauucgcggugcacuucu..........................   1       0
    ...........................................................................gaaacauucgcggugcacuucu..........................   5       0
    ...........................................................................gaaacauucgcggugcacuucuuu........................   1       0
    ............................................................................aaacauucgcggugcacu.............................   1       0
    ............................................................................aaacauucgcggugcacuu............................   1       0
    ............................................................................aaacauucgcggugcacuuc...........................   2       0
    ............................................................................aaacauucgcggugcacuucu..........................   523     0
    ............................................................................aaacauucgcggugcacuucuu.........................   4970    0
    ............................................................................aaacauucgcggugcacuucuuu........................   1468    0
    ............................................................................aaacauucgcggugcacuucuuuu.......................   56      0
    ............................................................................aaacauucgcggugcacuucuuuuuc.....................   1       0
    .............................................................................aacauucgcggugcacuucu..........................   2       0
    .............................................................................aacauucgcggugcacuucuu.........................   11      0
    .............................................................................aacauucgcggugcacuucuuu........................   16      0
    ..............................................................................acauucgcggugcacuucuu.........................   1       0
```
